# No Difference in the Rate of Change in Telomere Length or Telomerase Activity in HIV-Infected Patients after Three Years of Darunavir/Ritonavir with and without Nucleoside Analogues in the MONET Trial

**DOI:** 10.1371/journal.pone.0109718

**Published:** 2014-11-04

**Authors:** Ajantha Solomon, Surekha Tennakoon, Edwin Leeansyah, Jose Arribas, Andrew Hill, Yvon Van Delft, Christiane Moecklinghoff, Sharon R. Lewin

**Affiliations:** 1 Department of Infectious Diseases, Monash University and Alfred Health, Centre for Biomedical Research, Burnet Institute, Melbourne, Australia; 2 Center for Infectious Medicine, Department of Medicine, Karolinska Institutet, Karolinska University Hospital Huddinge, Stockholm, Sweden; 3 Hospital la Paz, IdiPAZ, Madrid, Spain; 4 Janssen, High Wycombe, United Kingdom; 5 Janssen, Tilburg, Netherlands; 6 Janssen, Neuss, Germany; Rush University, United States of America

## Abstract

**Objective:**

To determine whether nucleos(t)ide reverse transcriptase inhibitors (NRTI) contribute to an accelerated loss in telomere length (TL) in HIV-infected patients on antiretroviral therapy (ART).

**Design:**

Substudy of randomised controlled trial.

**Methods:**

Patients with HIV RNA <50 copies/mL on combination ART (n = 256) were randomised to darunavir/ritonavir (DRV/r) 800/100 mg once daily, either as monotherapy (n = 127) or with 2 NRTIs (n = 129) for up to 144 weeks. TL and telomerase activity was quantified on stored peripheral blood mononuclear cells (PBMC; n = 124) using quantitative real time PCR.

**Results:**

Patients in the sub-study had a mean age of 44 years and had received NRTI for a mean of 6.4 years (range 1–20 years). As expected, older patients have significantly shorter TL (p = 0.006), while women had significantly longer TL (p = 0.026). There was no significant association between TL and either the duration of prior NRTI treatment (p = 0.894) or the use of a PI versus NNRTI (p = 0.107). There was no significant difference between patients who continued or ceased NRTI in the mean change/year of TL or telomerase (p = 0.580 and 0.280 respectively).

**Conclusion:**

Continuation versus cessation of NRTI treatment was not associated with an accelerated loss in TL or telomerase activity.

## Introduction

Despite the great success of effective antiretroviral therapy (ART), HIV-infected individuals are at increased risk of age-related complications [1], [2]. Shorter telomere length (TL) has been associated with older age [3], cardiovascular disease [4] and lower survival in the general population [5], [6]. HIV infection has been associated with shorter TL in vivo [7]–[9] and inhibition of telomerase in vitro [10]. Factors contributing to shorter TL may potentially explain why HIV-infected individuals are at increased risk of age-related complications.

Telomerase is a DNA polymerase responsible for the maintenance of TL. It is a ribonucleoprotein enzyme complex containing a critical telomerase reverse transcriptase (RT) subunit which is required for addition of hexameric nucleotides to the telomeric regions. Nucleos(t)ide reverse transcriptase inhibitors (NRTI) that inhibit HIV RT also inhibit telomerase activity *in vitro* [11], [12] via inhibition of telomerase RT [13] and may potentially contribute to an accelerated decrease in TL. NRTIs could therefore potentially accelerate ageing in HIV-infected patients. In a cross sectional study of HIV-infected patients on ART, we recently demonstrated that a longer duration of NRTI treatment was associated with significantly shorter TL in peripheral blood mononuclear cells (PBMCs) [14].

The MONET trial [15] recruited patients who had HIV RNA less than 50 copies/mL while taking combination ART of two NRTI with either a protease inhibitor (PI) or a non-nucleoside reverse transcriptase inhibitor (NNRTI). The patients were taking different NRTI at the screening visit – the most common were tenofovir (TDF), abacavir (ABC) or zidovudine (ZDV), typically taken in combination with lamivudine (3TC) or emtricitabine (FTC). Patients were randomised to either continue their NRTI in combination with darunavir/ritonavir (DRV/r, at the 800/100 mg once daily dose) or to switch to DRV/r monotherapy, for up to 144 weeks.

The telomere sub-study of the MONET trial was designed to answer two questions: (1) whether, at the baseline visit, TL and telomerase activity correlated with the duration of prior NRTI treatment and (2) whether a switch to DRV/r monotherapy was associated with smaller reductions in TL over 144 weeks, compared to continued treatment with NRTI in the control arm.

## Methods

The design and main results of the MONET trial have been described previously [15]. Briefly, 256 patients with HIV RNA <50 copies/mL on combination ART (2 NRTI plus PI or NNRTI) were randomised to DRV/r 800/100 mg once daily, either as monotherapy (n = 127) or with 2 NRTIs (n = 129) for up to 144 weeks. All patients signed written informed consent at screening, and the trial was approved by local and national ethics committees. The study is registered at ClinicalTrials.gov (NCT00458302).

**Figure 1:**
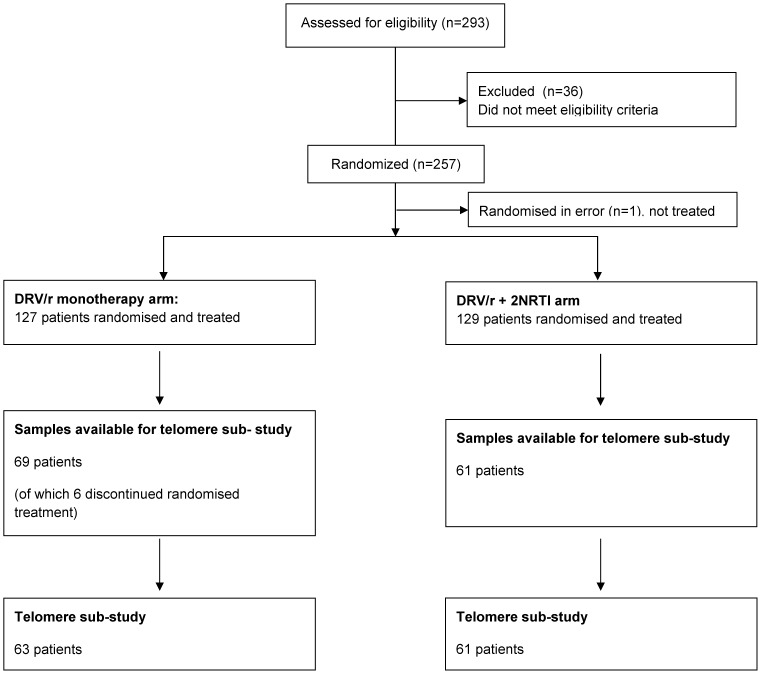
MONET trial- Telomere sub-study patient disposition.

The sub-study to measure TL and telomerase activity was conducted on stored samples after the trial had been completed. We measured TL and telomerase activity in PBMCs from 130 of the 256 randomised patients who had stored samples available. Six of the patients in the DRV/r monotherapy arm received NRTI during the study and were therefore excluded from the eligible population (n = 124) (Figure 1).

We used the same methods for sample analysis as previously described [14]. Briefly, TL was measured using real-time quantitative PCR; TL was expressed as a ratio to a single (S) copy housekeeping gene 36B4 (T/S ratio). Telomerase activity was measured using a real-time quantitative telomerase repeats amplification protocol (RQ-TRAP). Each patient was tested at baseline and at their last visit (either Week 48, Week 96, or Week 144).

All data on telomerase activity were log_10_ transformed, given the positive skew in the data. Multivariable linear regression was used to identify factors predictive of TL and telomerase activity at the baseline visit. We included the following predictive factors: treatment arm, age, sex, and years of prior NRTI treatment and use of a PI or NNRTI at baseline.

Given that patients had their last visit at different follow up times (either Week 48, 96 or 144), we calculated the annual change in TL and telomerase activity. The change from baseline in TL and log_10_ telomerase activity was analysed by treatment arm and for patients taking different NRTIs in the control arm – either TDF, ABC or ZDV. The Wilcoxon test was used to compare annual changes in TL and telomerase activity between the treatment arms.

## Results

The baseline characteristics of the 124 patients in this sub-study are shown in Table 1. The patients in the sub-study had a mean age of 44 years and 23 (18%) were female. They had received NRTI for a mean of 6.4 years (range 1–20 years). There were no statistically significant differences in the baseline characteristics across the two treatment arms, except for sex where there were more females in the DRV/r monotherapy arm (24%) than the DRV/r + 2NRTI arm (10%; p = 0.05). Of the 124 patients in the sub-study, 73% had follow up data to Week 144, 15% to Week 96 and 12% to Week 48. The characteristics of these patients were similar to the patients not included in the sub-study (n = 126) because stored PBMC samples were not available (data not shown).

**Table 1:** Baseline characteristics, telomere length and telomerase activity by treatment arm.

Baseline characteristic	DRV/r + 2NRTIs, n = 61	DRV/r, n = 63
Age, years	44.1 (10.3)	43.7 (9.9)
Sex: n (%) female	6 (10%)	15 (24%)
Race: n (%) Caucasian	58 (95%)	58 (92%)
CD4 count, cells/uL	667 (311)	624 (270)
Nadir CD4 count, cells/uL	257 (122)	260 (116)
Duration of prior NRTI, years	5.8 (3.3)	6.8 (3.9)
Use of PI at screening: n (%)	36 (59%)	36 (52%)
Baseline telomere length (T/S ratio)	1.27 (0.38)	1.54 (0.58)
Telomere length at last visit (T/S ratio)	1.17 (0.43)	1.46 (0.55)
Annual change in T/S ratio	−0.039 (0.12)	−0.037 (0.17)
Baseline Telomerase activity (log_10_)	1.54 (0.36	1.50 (0.36)
Telomerase activity at last visit (log_10_)	1.54 (0.36)	1.43 (0.38)
Annual change in telomerase activity (log_10_)	+0.005 (0.49)	−0.068 (0.46)

T/S ratio: TL was expressed as a ratio to a single (S) copy housekeeping gene 36B4 (T/S ratio). All parameters are shown as mean (SD), unless otherwise stated.

Factors associated with baseline TL and telomerase activity were analysed by multivariable analysis (Table 2). As expected, older patients has significantly shorter TL (p = 0.006), while women had significantly longer TL (p = 0.026). There was no significant association between TL and either the duration of prior NRTI treatment (p = 0.894) or the use of a PI versus NNRTI (p = 0.107). In the multivariable analysis, TL was significantly longer in PBMC from patients enrolled in the DRV/r monotherapy arm, compared to the DRV/r + 2NRTIs arm (p = 0.008). The reason for this baseline difference was unclear – the longer TL in the DRV/r monotherapy arm was also observed when only men were included in the analysis (p = 0.02). None of the baseline predictors showed significant associations with telomerase activity.

**Table 2:** Multivariable analysis of TL and log_10_ (Telomerase activity) at baseline.

		Telomere length		Telomerase activity	
Predictor	level	Adjusted coefficient (95% CI)	p value	Adjusted coefficient (95% CI)	p value
Treatment arm	DRV/r vs control	+0.23 (+0.06, +0.41)	0.008	+0.04 (−0.17, +0.09)	0.544
Age	per year older	−0.01 (−0.02, −0.003	0.006	+0.003 (−0.002, +0.011)	0.18
Sex	female vs male	+0.26 (+0.03, +0.49)	0.026	−0.118 (−0.28, +0.05)	0.175
Years of prior NRTI	per year of NRTI	−0.001 (−0.26, +0.23)	0.894	+0.007 (−0.01, +0.02)	0.402
Use of PI at screening	use of PI	−0.14 (−0.31, +0.03)	0.107	−0.020 (−0.11, +0.15)	0.761

Multivariable linear regression was used to identify factors predictive of TL and telomerase activity at the baseline visit.

In the DRV/r monotherapy arm, the mean (standard deviation, SD) TL (T/S ratio) declined from 1.54 (0.58) at baseline to 1.46 (0.55) at the end of treatment (mean change/year  = −0.04). In the DRV/r +2NRTIs arm, the mean TL declined from 1.27 (0.38) at baseline to 1.17 (0.43) at the end of treatment (mean change/year  = −0.04). There was no significant difference in the mean change/year of TL between arms (p = 0.580).

We then analysed annual changes in TL in PBMC from patients enrolled in the DRV/r +2NRTI arm according to the NRTIs taken. The mean (SD) annual change in TL in patients taking TDF was −0.05 (0.097)/year (n = 23); ABC was −0.05 (0.154)/year (n = 22); and ZDV was −0.03 (0.091)/year (n = 14). There were two patients taking other NRTIs, who were not included in this analysis.

The changes from baseline in log_10_ telomerase activity are also shown in Table 1. Overall, the mean log_10_ telomerase activity was 1.51 at baseline, with no differences between arms (p = 0.239). The mean (SD) change in telomerase activity from baseline was −0.07 (0.46) log_10_ in the DRV/r monotherapy arm and +0.01 (0.49) in the DRV/r +2NRTIs arm, with no significant difference between the arms (p = 0.280). The mean (SD) change in telomerase activity was then analysed in the DRV/r +2NRTI arm, for patients taking different NRTIs. The mean annual log_10_ change in patients taking TDF, ABC and ZDV was −0.17 (0.44), +0.17 (0.41) and +0.09 (0.64) respectively.

## Discussion

In the MONET trial, continuation compared to cessation of NRTI was not associated with an accelerated decrease in TL or change in telomerase activity. As expected TL was influenced by both age and sex [3].

This was the first prospective study to assess change in TL and telomerase activity following cessation of NRTI. Although this study was performed as a retrospective sub study of the original parent study, the study was randomised which is a strength. All previous studies assessing the relationship of TL and telomerase activity to NRTI in HIV infection have either been cross sectional [14] or not specifically examined the effects of NRTI as a component of ART [7]–[9]. Therefore, the true contribution of NRTI to TL and telomerase activity has never been defined.

There are several possible explanations for our findings. First, any change in TL or telomerase activity following NRTI treatment may be non–reversible. Second, decrease in TL could be slow and therefore we could have been underpowered to detect a difference given the duration of follow up. Third, measurement of TL and telomerase activity in total PBMC may have remained stable while there may have been changes in the TL in specific T-cell subsets or the proportion of different T cell subsets such as memory and effector CD4 and CD8 T-cells. Finally, it is possible that those randomised to the monotherapy arm of DRV/r could have potentially had greater residual virus replication in tissue sites such as lymph node [16] which may have led to a reduction of telomere length and therefore would have obscured any beneficial effects of cessation of NRTI.

The relationship between telomerase activity and NRTI was different in this longitudinal study was different to our previous report [14] where we showed in a cross sectional study of HIV-infected patients receiving ART, that telomerase activity was significantly higher in patients who were receiving a regimen that didn't include an NRTI. In our previous study, patients had been off an NRTI for a median of 48 weeks, a similar duration to the follow up period off NRTI in the current study. The different findings to the current study may be because the prior study was a cross sectional analysis and there may have been other confounders that were not accounted for in the two patient groups. However, it is interesting to note that in the previous publication, we quantified TL and telomerase in a small number of patients (n = 6) who had matched longitudinal samples ie prior to and following cessation of an NRTI, and in these patients we saw no significant change in telomerase (or TL) as we describe in this current study.

There were several limitations to this study. First, the sample size was limited but only 130 of the 256 patients in the MONET trial had stored samples available for analysis of TL. Therefore, the inability for us to detect a difference between the two arms may have been a consequence of the sample size and consequently the limited power to detect differences between the treatment arms [17]. Second, the overall duration of prior NRTI treatment was recorded in the database, but not the time that the patient was taking each individual NRTI. If the NRTIs had different effects on TL, as suggested in vitro [14], this could not be measured in the current analysis. Third, there was a baseline imbalance in TL between the treatment arms which was not accounted for by differences in sex. Finally, the duration of follow up differed for the participants but we adjusted for this by expressing TL and telomerase activity as a change over time.

In conclusion, we were unable to demonstrate a difference in annual decline in TL or telomerase in HIV-infected patients who had received long term NRTI-based ART and then ceased NRTI. Further studies are required to determine the complex relationship between NRTI, HIV and TL in vivo and the association of TL with age-related complication in HIV infection.
